# Allelic expression analysis of the osteoarthritis susceptibility locus that maps to *MICAL3*

**DOI:** 10.1186/1471-2350-13-12

**Published:** 2012-03-02

**Authors:** Madhushika Ratnayake, Louise N Reynard, Emma VA Raine, Mauro Santibanez-Koref, John Loughlin

**Affiliations:** 1 Musculoskeletal Research Group, Institute of Cellular Medicine, Newcastle University, Newcastle, UK; 2 Institute of Genetic Medicine, Newcastle University, Newcastle, UK

## Abstract

**Background:**

A genome-wide association scan with subsequent replication study that involved over 67,000 individuals of European ancestry has produced evidence of association of single nucleotide polymorphism rs2277831 to primary osteoarthritis (OA) with a P-value of 2.9 × 10^-5^. rs2277831, an A/G transition, is located in an intron of *MICAL3*. This gene is located on chromosome 22q11.21 and the association signal encompasses two additional genes, *BCL2L13 *and *BID*. It is becoming increasingly apparent that many common complex traits are mediated by *cis*-acting regulatory polymorphisms that influence, in a tissue-specific manner, gene expression or transcript stability.

**Methods:**

We used total and allelic expression analysis to assess whether the OA association to rs2277831 is mediated by an influence on MICAL3, BCL2L13 or BID expression. Using RNA extracted from joint tissues of 60 patients who had undergone elective joint replacement surgery, we assessed whether rs2277831 correlated with allelic expression of either of the three genes by: 1) measuring the expression of each gene by quantitative PCR and then stratifying the data by genotype at rs2277831 and 2) accurately discriminating and quantifying the mRNA synthesised from the alleles of OA patients using allelic-quantitative PCR.

**Results:**

We found no evidence for a correlation between gene expression and genotype at rs2277831, with P-values of 0.09 for *BCL2L13*, 0.07 for *BID *and 0.33 for *MICAL3*. In the allelic expression analysis we observed several examples of significant (p < 0.05) allelic imbalances, with an allelic expression ratio of 2.82 observed in *BCL2L13 *(P = 0.004), 2.09 at *BID *(P = 0.001) and the most extreme case being at *MICAL3*, with an allelic expression ratio of 5.47 (P = 0.001). However, there was no correlation observed between the pattern of allelic expression and the genotype at rs2277831.

**Conclusions:**

In the tissues that we have studied, our data do not support our hypothesis that the association between rs2277831 and OA is due to the effect this SNP has on *MICAL3, BCL2L13 *or *BID *gene expression. Instead, our data point towards other functional effects accounting for the OA associated signal.

## Background

Osteoarthritis (OA) is a common disease of the synovial joint characterized by cartilage loss and is often accompanied by alteration in the normal function of other tissues of the articulating joint [1]. Based on epidemiological studies, the disease is known to have a major genetic component, although elucidating this has proven challenging [2]. This is a reflection of the heterogeneity associated with the disease, with severe OA being a clinical end-point reached from a multitude of starting points. Genome-wide association scans (GWASs) are now being employed as powerful, objective tools for mapping susceptibility loci of complex diseases including OA [3,4]. The resulting signals originate from regions of the genome that do not harbour obvious OA candidates and an example of this came from stage one of the recently published arcOGEN GWAS [5]. In an analysis of over 67,000 individuals of European descent this study identified, amongst other signals, an association to SNP rs2277831 (A/G), with a P-value of 2.9 × 10^-5 ^and an odds ratio for allele G of 1.07 in knee and/or hip OA.

rs2277831 is located within intron 32 of the *MICAL3 *gene, which codes for the microtubule-associated monoxygenase, calponin and LIM domain containing 3 protein. The MICAL3 protein is thought to be involved in the vesicle transport system in mammalian cells through the interaction with Rabs, which are small GTPases [6,7]. Two additional genes map close by and are encompassed by the association signal. These are *BCL2L13 *(also known as *BCL-Rambo*), which codes for the BCL2-like 13 (apoptosis facilitator) protein, and *BID*, which codes for the BH3 interacting domain death agonist protein. The BCL2L13 protein has structural homology to BCL2, and is thought to induce apoptosis through the activation of caspase-3 [8]. The BID protein is also proapoptotic and is cleaved by Caspase-8. The cleaved form translocates to the mitochondria where it forms heterodimers with BAX or BCL2 and triggers the release of cytochrome *c*, leading to the activation of apoptotic pathways [9]. Scrutinizing public databases reveals that *MICAL3 *gene is expressed in various tissue types, including adipose tissue, blood, bone, brain, cartilage, heart, liver, kidney and muscle. *BCL2L13 *and *BID *expression are also observed in many tissue types.

As the number of susceptibility loci that are identified for common human diseases increases, it is becoming clear that many, if not the majority, of associated alleles contribute to disease risk by acting as expression quantitative trait loci (eQTLs), influencing the expression or stability of a transcript [10,11]. In the majority of cases eQTLs act in an organ or even tissue specific manner. It is important therefore to study RNA extracted from tissues relevant to the disease when assessing the potential effect of eQTLs.

We hypothesized that the OA association to rs2277831 may be marking an eQTL. To assess this we have measured the overall expression of *BCL2L13, BID *and *MICAL3 *using real time reverse transcription PCR and stratified the expression by the genotype at rs2277831. We also tested for allelic expression imbalance of *BCL2L13, BID *and *MICAL3 *using transcript SNPs and assays that can accurately discriminate and quantify the mRNA synthesised from each allele. We examined RNA extracted from the joint tissues of patients undergoing elective joint replacement surgery.

## Methods

### Patients

Joint tissues from 60 individuals who had undergone elective joint replacement of the hip (total hip replacement, THR) or the knee (total knee replacement, TKR) were obtained and nucleic acids were extracted as described previously [12]. The tissues collected were cartilage, fat pad, synovium, cancellous bone and osteophyte. Of these 60 individuals, 59 patients had OA and one patient did not have clinical OA but had undergone a neck-of-femur (NOF) fracture. The Newcastle and North Tyneside research ethics committees granted ethical approval for the collection (REC reference number 09/H0906/72) and informed consent was obtained from each donor.

### cDNA synthesis

2.2 μg of RNA was DNase treated with Turbo DNase (2 units; Ambion) according to the manufacturer's protocol. Reverse transcription was carried out using the SuperScript First-Strand cDNA Synthesis kit (Invitrogen) according to the manufacturer's instructions. The synthesised cDNA was PCR amplified using primers located in different exons, that is, the amplimer spans one or more introns if the target template were DNA. The use of such primers distinguishes cDNA products from any products that may result from residual genomic DNA contamination. The PCR products were electrophoresed through a 3% weight/volume agarose gel containing ethidium bromide. Only cDNA samples that produced appropriate sized product were taken forward for allelic expression imbalance analysis.

### Qualitative gene expression

The expression of *BCL2L13, BID *and *MICAL3 *in cDNA synthesised from joint tissue RNAs was assessed using the following primers: *BCL2L13*, 5'GGAATTGACAAGACGTGGTC3' and 5'GCTTTCTGTGTGCCAACTCTGG3'; *BID*, 5'AGACATCATCCGGAATATTGC3' and 5'TGTGAAAGACATCACGGAGC3'; *MICAL3*, 5'GTGGACTCTGGAAAGCACAG3' and 5'CAGCTTGGCATTCAGTTCCTC3'.

### Quantitative gene expression analysis

RNA from cartilage tissue from 32 patients was extracted and cDNA was synthesised as described above. Quantitative gene expression of *BCL2L13, BID *and *MICAL3 *was then measured by real time reverse transcription PCR using custom designed TaqMan primers and probes (Integrated DNA Technologies; Additional file 1). The reactions were performed in triplicate on an ABI PRISM 7900HT Sequence Detection System. The expression was measured relative to the housekeeping genes *18S, GAPDH *and *HPRT1*. The delta Ct value was calculated using the formula: Delta Ct = Ct (test gene) - Ct (average of control genes).

Cartilage DNA from the 32 patients was used to genotype the patients for the associated SNP rs2277831, as described below. For each gene, the delta Ct values for each patient were plotted against the rs2277831 genotype and linear regression was used to determine if gene expression relative to genotype differed significantly from the null.

### SNP genotyping

**Figure 1:**
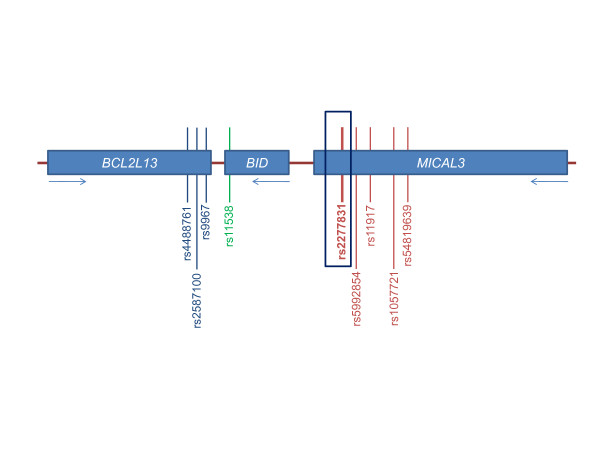
**Relative positions of the selected transcribed SNPs**. The OA associated variant rs2277831 is boxed. Arrows indicate the direction in which each gene is transcribed.

For the allelic expression analysis, we studied eight transcript SNPs, three in *BCL2L13*, one in *BID *and four in *MICAL3 *(Figure 1). The linkage disequilibrium (LD) between rs2277831 and the transcript SNPs apart from rs5992854 was low (Table 1 and Additional file 2). Genotyping of these SNPs, and of the OA associated SNP rs2277831, was carried out by restriction fragment length polymorphism (RFLP) analysis. Initially 50 ng of genomic DNA from patient tissue was PCR amplified in a 15 μl reaction using SNP specific forward and reverse primers (Additional file 3). The PCR products were then incubated with 5 μl of a digestion mix containing the corresponding restriction enzyme (5 U; New England Biolabs; Additional file 3), 1.5 μl of the recommended NEB buffer (10 x), BSA (100 x) where required and water. Digestion reactions were incubated for 3 hours at the optimum temperature for the restriction enzyme. The digested products were then electrophoresed through agarose as described above.

**Table 1:** The eight transcript SNPs studied in this report

SNP	Position on chr. 22 on NCBI genome build 37.3	Alleles	MAF*	Gene	Location of SNP in gene	Pairwise linkage disequilibrium relative to the OA associated SNP rs2277831	
						
						D'	r^2^
rs4488761	18209613	A/G	0.46	*BCL2L13*	3'UTR/exonic^^^	0.11	0.00

rs2587100	18210704	C/G	0.40	*BCL2L13*	3'UTR	0.02	0.00

rs9967	18211205	C/T	0.38	*BCL2L13*	3'UTR	0.21	0.01

rs11538	18220831	T/C	0.18	*BID*	3'UTR/exonic^^^	0.12	0.00

rs5992854	18300240	T/C	0.32	*MICAL3*	exonic	1.00	0.71

rs11917	18343011	G/T	0.41	*MICAL3*	3'UTR	0.76	0.11

rs1057721	18343843	G/A	0.41	*MICAL3*	3'UTR	0.78	0.13

rs4819639	18347127	C/T	0.29	*MICAL3*	3'UTR	0.74	0.06

*Minor allele frequency in Europeans (dbSNP http://www.ncbi.nlm.nih.gov/projects/SNP/^^^Multiple predicted splice isoforms result in different potential locations in the transcript

### Allelic expression analysis

Allelic expression imbalance was assessed using Sequenom assays for six of the eight transcript SNPs (rs4488761, rs2587100, rs11917, rs9967, rs1057721, rs4819639) and assessed using quantitative real time PCR genotyping assays for the remaining two SNPs (rs5992854 and rs11538). Patients who were heterozygous for one or more of the eight SNPs were selected for allelic expression imbalance analysis.

Sequenom analysis involves a single-base primer extension into the SNP of interest using dideoxyNTPs (ddNTPs). The two alleles at the SNP are then distinguished using the difference in mass between the ddNTPs that are incorporated into the extension primer. Sequenom assays (Additional file 4) were designed using the online 'MySequenom Genotyping Tools' https://www.mysequenom.com/Tools. The extension primer used for one SNP typically differs in mass from the extension primers used for other SNPs, thus enabling multiplexing. We prepared two multiplex reactions for the six SNPs. DNA and the respective cDNA were amplified separately using the forward and reverse primers listed in Additional file 4 in a 15 μl multiplex reaction. The reactions were performed in four replicates. A 5 μl aliquot of each PCR product was then incubated with 0.5 U of Shrimp alkaline phosphatase (SAP; Sequenom) at 37°C for 40 minutes and 85°C for 5 minutes. The multiplex extension reaction was then carried out in a 9 μl volume with 10 × iPLEX buffer, iPLEX termination mix, extension primer mix (primers at 7 to 14 μM each in a multiplex pool) and iPLEX enzyme (Sequenom). The extension reaction products were then purified with SpectroCLEAN (Sequenom), arrayed on to spots on a SpectroCHIP and analysed in a Sequenom MassARRAY Analyzer. The SpectroTYPER software (Sequenom) generates a peak for each extension fragment at the expected mass, which corresponds to the amount of each allele in the sample analysed.

Quantitative real time PCR genotyping assays are standard real time assays except that they employ a probe (FAM or VIC labelled) specific to each of the two alleles at a SNP. Readymade TaqMan genotyping assays for rs5992854 and rs11538 were purchased from Applied Biosystems. Real time PCR was carried out according to the manufacturer's instructions on an ABI PRISM 7900HT Sequence Detection System. The allelic ratios were calculated using the formula (2^-FAM Ct)/(2^-VIC Ct).

For each Sequenom and real time PCR assay, four replicates were performed per DNA and per cDNA. For each assay, the ratios between the amounts of each allele in every sample were calculated for genomic DNA and cDNA. For each sample the average allelic ratio for genomic DNA, which represents the 1:1 ratio, was then used to normalise the average cDNA ratio to generate a normalised allelic ratio using the following formula:

$$\text{Normalised allelic ratio}=\frac{\text{Average allelic ratio of cDNA}}{\text{Average allelic ratio of gDNA}}$$

Tissue samples were considered to show an allelic imbalance if the fold difference in expression from the two alleles was 20% or greater. A two-tailed Mann-Whitney exact test was performed to compare the distribution between the allelic ratios of the DNA replicates and the cDNA replicates. A Kruskal-Wallis test was performed to assess the association between allelic expression and the genotype at rs2277831.

## Results

Expression of *BCL2L13, BID *and *MICAL3 *was observed in the five joint tissues analysed; cartilage, fat pad, synovium, cancellous bone and osteophyte (data not shown).

**Figure 2:**
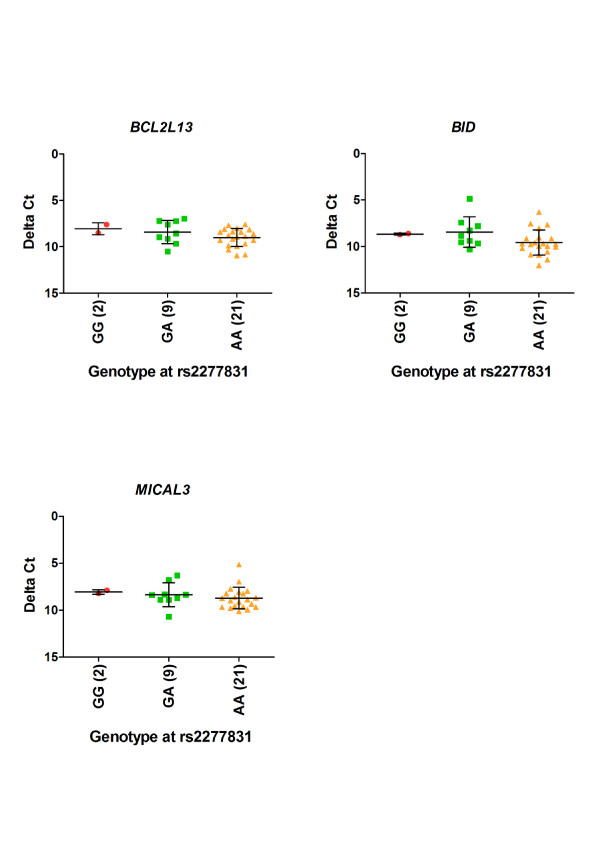
**Expression in the cartilage of 32 OA patients of *BCL2L13, BID *and *MICAL3 *stratified by genotype at the OA associated SNP rs2277831**. The data points represent the average of the three replicates for each sample. The expression of (A) *BCL2L13*, (B) *BID *and (C) *MICAL3 *was compared to the average of the housekeeping genes and the delta Ct values were plotted against the genotype at the associated SNP rs2277831. The horizontal bars represent the mean plus the standard deviation. The genotype at rs2277831 did not correlate with gene expression with P-values of 0.09 for *BCL2L13*, 0.07 for *BID *and 0.33 for *MICAL3*.

We quantitatively measured the expression of *BCL2L13, BID *and *MICAL3 *in cartilage RNA in 32 OA patients and stratified the expression data by genotype at the OA associated SNP rs2277831. Two patients were homozygous GG, 9 were heterozygous and 21 were homozygous AA. There was no significant correlation between genotype at rs2277831 and the expression of *BCL2L13, BID *or *MICAL3*, with P-values of 0.09, 0.07 and 0.33, respectively (Figure 2, Additional file 5).

Correlating genotype with overall gene expression is vulnerable to the natural fluctuation in gene expression, and as such is liable to generating false-negative results. Directly testing for allelic expression differences can overcome this. We therefore subsequently identified 33 patients who were heterozygous at one or more of the eight transcript SNPs (Table 2). Thirty-two of the 33 were OA patients whereas patient 42 had undergone joint surgery due to a hip fracture. For two of the OA patients we had collected more than one tissue type: fat pad and synovium for patient 52, cancellous bone and osteophyte for patient 60. For all other patients we had collected only one tissue type per patient.

**Table 2:** Allelic expression analysis for 33 patients

						*BCL2L13*				*BID*						*MICAL3*				
				
**Patient no**.	Sex	Age at surger y (years)	Tissue*	rs4488761		rs2587100		rs9967		rs11538		rs2277831	rs5992854		rs11917		rs1057721		rs4819639	
				
				Gen^^^	AER† (A/G)	Gen^^^	AER† (C/G)	Gen^^^	AER† (C/T)	Gen^^^	AER† (T/C)	Gen^^^	Gen^^^	AER† (T/C)	Gen^^^	AER† (T/G)	Gen^^^	AER† (G/A)	Gen^^^	AER† (C/T)
1	F	55	Cart (K)	AG	1.04	CG	**0.67**	CT	**0.71**	TC	0.90	GG	CC	-	GG	-	AA	-	CC	-

33	M	74	Cart (K)	AG	1.15	CG	1.03	CT	0.91	TT	-	GG	CC	-	GG	-	AA	-	CC	-

3	M	82	Cart (K)	AA	-	CG	0.83	TT	-	TC	1.10	GA	TC	1.13	GG	-	AA	-	CT	0.96

4	M	57	Cart (K)	AG	0.95	CG	0.87	CT	1.09	TC	1.06	GA	TC	**0.56**	TG	**1.30**	GA	**2.10**	CT	**1.38**

34	F	57	Cart (K)	AA	-	GG	-	TT	-	TT	-	GA	TC	1.14	TG	0.88	GA	0.84	CT	0.90

35	M	58	Cart (K)	AA	-	CG	**0.78**	TT	-	TT	-	GA	TC	**1.30**	TG	1.11	GA	0.87	CT	1.02

36	M	71	Cart (K)	GG	-	GG	-	CC	-	TT	-	GA	TC	1.02	GG	-	AA	-	CC	-

37	F	72	Cart (K)	AG	1.09	CG	0.91	CT	1.15	TC	**1.54**	AA	TT	-	TG	**1.51**	GA	0.85	CT	0.87

38	M	64	Cart (K)	AG	0.91	CG	1.01	CT	1.09	TT	-	AA	TC	**5.47**	TT	-	GG	-	CC	-

39	M	76	Cart (K)	GG	-	CG	0.88	CT	**1.33**	TT	-	AA	TT	-	TG	1.01	GA	**1.51**	CT	0.82

13	F	66	Cart (K)	AG	**1.40**	CG	**2.82**	CT	**0.57**	TT	-	AA	TC	**1.22**	TT	-	GG	-	TT	-

26	F	67	Cart (H)	AG	**1.67**	CG	**1.98**	CT	1.01	TT	-	AA	TC	0.83	TT	-	GG	-	TT	-

40	F	76	Cart (H)	AG	1.04	CG	0.99	CT	**1.22**	TC	1.01	AA	TT	-	TG	**0.76**	GA	1.14	CT	**1.49**

41	F	50	Cart (H)	GG	-	CG	0.94	CC	-	TT	-	AA	TT	-	TT	-	GG	-	CT	0.83

42	M	83	Cart (H)	AG	0.97	CG	1.00	CT	1.18	TC	0.96	AA	TC	1.03	TG	1.00	GA	1.16	CT	0.93

43	M	62	Fat pad	AG	1.06	CG	1.00	CT	1.01	TC	0.94	GG	CC	-	TG	1.09	GA	**1.39**	CC	-

44	M	57	Fat pad	AG	1.01	GG	-	CT	**0.80**	TT	-	GG	TT	-	GG	-	AA	-	CC	-

45	M	76	Fat pad	GG	-	GG	-	CC	-	TT	-	GA	TC	**1.92**	GG	-	AA	-	CC	-

46	M	79	Fat pad	GG	-	GG	-	CC	-	TT	-	GA	TC	0.96	TG	1.03	GA	1.13	CT	1.02

47	M	58	Fat pad	AG	0.99	CC	-	TT	-	TT	-	GA	TT	-	GG	-	AA	-	CC	-

48	M	58	Fat pad	AG	1.02	CG	1.00	CT	**1.36**	TT	-	AA	TT	-	TG	**0.80**	GA	**0.63**	CT	1.09

49	F	42	Fat pad	AG	1.05	CG	0.94	CT	1.15	TT	-	AA	TT	-	TG	**2.13**	GA	**1.22**	CT	0.92

50	M	71	Fat pad	GG	-	CG	1.00	CT	1.13	TT	-	AA	TT	-	TG	**1.27**	GA	**1.23**	CC	-

51	F	67	Fat pad	AG	0.95	CG	1.05	CT	0.95	TT	-	AA	TC	**0.75**	TT	-	GG	-	TT	-

52	F	78	Fat pad	AG	1.15	CG	0.89	CT	0.92	TT	-	AA	TT	-	GG	-	AA	-	CC	-

53	M	74	Syn	AG	**0.80**	GG	-	CT	**1.33**	TT	-	GG	CC	-	GG	-	AA	-	CC	-

54	M	62	Syn	AG	0.99	CG	0.85	CT	**1.23**	TT	-	GA	TC	1.12	GG	-	AA	-	CC	-

55	M	67	Syn	AG	0.97	CG	0.98	CT	**1.32**	TC	0.83	GA	TC	0.81	TG	**0.57**	GA	**0.75**	CT	**1.24**

56	M	60	Syn	AG	1.11	GG	-	CT	1.14	TT	-	AA	TT	-	TG	1.19	GA	**1.32**	CC	-

57	M	69	Syn	AG	0.85	CG	**1.98**	CT	**0.72**	TC	**2.09**	AA	TT	-	TT	-	GG	-	CT	**0.77**

58	F	54	Syn	AG	0.90	CG	1.16	CT	1.19	TC	**1.56**	AA	TT	-	TG	**1.85**	GA	**1.89**	CC	-

52	F	78	Syn	AG	1.00	CG	0.95	CT	1.02	TT	-	AA	TT	-	GG	-	AA	**-**	CC	-

59	F	72	Can bn	AA	-	CG	1.10	TT	-	TC	1.14	GA	TC	0.96	TG	0.86	GA	**1.23**	CT	1.07

60	M	86	Can bn	GG	-	GG	-	CC	-	TT	-	AA	TT	-	TG	**1.43**	GA	1.10	CC	-

60	M	86	OP	GG	-	GG	-	CC	-	TT	-	AA	TT	-	TG	**1.27**	GA	**1.31**	CC	-

*Cart (H), hip cartilage; Cart (K), knee cartilage; Syn, synovium; Can bn, cancellous bone;

OP, osteophyte

^^^Gen, genotype

†AER, allelic expression ratio

Bold text denotes allelic expression imbalance (AEI) at the 20% threshold.

**Figure 3:**
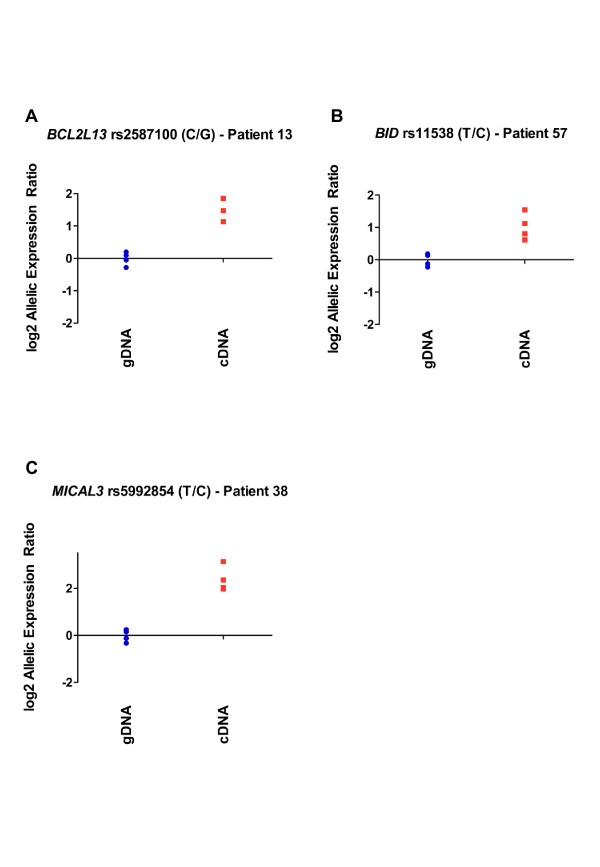
**Allelic expression imbalance in patients who showed the largest allelic ratios at *BCL2L13, BID *and *MICAL3***. Data points represent log2 of the normalised allelic ratio of genomic or cDNA for each replicate. A value of 0 on the Y-axis denotes an allelic ratio of 1:1. (A) Patient 13 demonstrated a 2.82 fold greater expression of the C allele relative to the G allele (P = 0.004) at *BCL2L13 *SNP rs2587100, (B) patient 57 demonstrated a 2.09 fold greater expression of the T allele relative to the C allele (P = 0.001) at *BID *SNP rs11538, (C) patient 38 demonstrated a 5.47 fold greater expression of the T allele relative to the C allele (P = 0.001) at *MICAL3 *SNP rs5992854.

Twenty-eight of the 33 patients were heterozygous for one or more of the three *BCL2L13 *transcript SNPs. Twelve of the 28 demonstrated allelic expression imbalance (AEI) when applying the arbitrary threshold of a 20% or greater difference in expression between alleles (data in bold in Table 2). Of these 12 patients, three were heterozygous for the associated SNP rs2277831 (patients 35, 54 and 55), three were homozygous GG at rs2277831 and six were homozygous AA at rs2277831. AEI at *BCL2L13 *did not therefore correlate with genotype at rs2277831. The largest allelic ratio observed at *BCL2L13 *was 2.82 (P = 0.004) at transcript SNP rs2587100 for patient 13 (Figure 3), who is homozygous AA at rs2277831.

Eleven patients were heterozygous for the *BID *transcript SNP rs11538. Three of the eleven demonstrated AEI at the 20% threshold, patients 37, 57 and 58 (data in bold in Table 2). There was no correlation between genotype at rs2277831 and AEI at *BID*, with all three patients being homozygous AA at this SNP. The largest allelic ratio observed at *BID *was 2.09 (P = 0.001) in patient 57 (Figure 3).

Twenty-seven patients were heterozygous for one or more of the four *MICAL3 *transcript SNPs. Nineteen of the 27 patients demonstrated AEI at the 20% threshold (data in bold in Table 2). Of these 19, five were heterozygous for the associated SNP rs2277831 (patients 4, 35, 45, 55 and 59), one was homozygous GG at rs2277831 and 13 were homozygous AA. AEI at *MICAL3 *did not therefore correlate with genotype at rs2277831. The largest allelic ratio observed at *MICAL3*, and at any of the three genes studied, was 5.47 (P = 0.001) at transcript SNP rs5992854 for patient 38 (Figure 3), who is homozygous AA at rs2277831.

**Figure 4:**
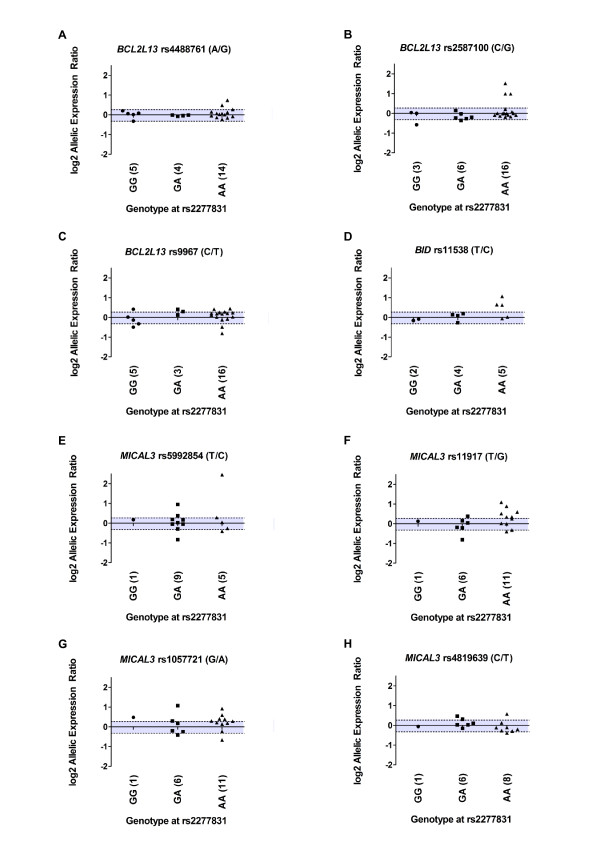
**Allelic expression imbalance in patients at the eight transcript SNPs studied**.

We noted that in some individuals who were heterozygous for more than one transcript SNP from a particular gene, the AEI pattern observed was not always consistent between the transcript SNPs. For example, for *BCL2L13 *patient 1 shows AEI at rs2587100 (0.67) and at rs9967 (0.71), but not at rs4488761 (1.04). *BCL2L13 *has 14 transcript isoforms and rs4488761 exists in nine of these whereas, rs2587100 and rs9967 exist in five. Such variability could account for such AEI differences. We therefore plotted the average allelic expression ratios for the heterozygotes at the eight transcript SNPs and stratified the data by genotype at rs2277831 (Figure 4). At any of the transcribed SNPs, the Kruskal-Wallis test showed no significant evidence of a difference in the allelic expression ratios between the different genotypes at rs2277831 (P-values of 0.45 at rs4488761, 0.11 at rs2587100, 0.20 at rs9967, 0.15 at rs11538, 0.88 at rs5992854, 0.35 at rs11917, 0.41 at rs1057721 and 0.53 at rs4819639).

The averaged log2 of the normalised allelic ratios are shown for each patient, categorised according to the genotype at the associated SNP, rs2277831. The shaded area represents a 20% fold difference in expression between the two alleles

Overall, we have studied hip cartilage, knee cartilage, fat pad, synovium, cancellous bone and osteophyte and although we observed AEI for one or more of the tissues in each gene, the AEI does not correlate with genotype at the OA associated SNP rs2277831.

## Discussion

It has become increasingly apparent that most common traits are influenced by large numbers of alleles that individually have small effects [13]. It is also becoming clear that many if not the majority of complex trait alleles influence the expression of the gene by modulating transcription or transcript stability [11]. In OA, the *GDF5 *SNP rs143383 is a very good example of this. *GDF5 *codes for growth and differentiation factor 5, an extracellular signalling molecule that participates in the development and maintenance of the synovial joint. rs143383 is a T to C transition located in the 5' untranslated region of *GDF5 *and it shows compelling association to OA in European and Asian cohorts (reviewed in [2]). Functional studies have suggested that rs143383 may itself be the polymorphism influencing OA susceptibility at this locus, with the OA associated T allele mediating reduced *GDF5 *transcription relative to the C allele in all joint tissues so far tested [14]. We therefore hypothesised that the recently reported knee and hip OA association signal at chromosome 22q11.21, detected at SNP rs2277831, may mediate its effect on OA susceptibility by modulating the expression of one or more of the three genes from within the signal. We chose to focus our expression studies on joint tissues from patients who had undergone joint replacement surgery rather than a tissue unrelated to OA, such as blood.

We first looked at overall expression of *BCL2L13, BID *and *MICAL3 *in cartilage tissue RNA stratified by genotype at rs2277831, with no significant correlations observed.

We subsequently tested each gene for the activity of *cis*-regulatory polymorphisms by measuring AEI. Our results revealed that AEI was common and was not restricted to a particular tissue type. If our hypothesis were correct, namely that the OA associated G allele of rs2277831 was a marker for altered expression of one or more of the three genes tested then we would predict that a heterozygote for this SNP would tend to demonstrate AEI. However, we did not observe any correlation between genotype at rs2277831 and AEI, such that an individual heterozygous for rs2277831 was no more likely to demonstrate AEI at one of the three genes than an individual homozygous at this SNP.

## Conclusions

Overall our study reveals that *BCL2L13, BID *and *MICAL3 *are subject to *cis*-acting polymorphism/s that influence the expression of the genes in the joint tissues of patients who have undergone surgery. Our data do not however support a role for such *cis*-regulation at these genes as accounting for the OA association signal at chromosome 22q11.21. Other functional effects are presumably involved.

## Competing interests

The authors declare that they have no competing interests.

## Authors' contributions

MR carried out gene expression analyses, genotyping, allelic expression experiments, performed the analyses and drafted the manuscript. LNR participated in the interpretation of the results and reviewed the manuscript. EVAR carried out tissue grinding and DNA and RNA extractions. MSK participated in the statistical analyses, discussing results, supervising the project and helped draft the manuscript. JL participated in the study design and coordination, discussing results, supervising the project and co-drafted the manuscript. All authors read and approved the final manuscript.

## Pre-publication history

The pre-publication history for this paper can be accessed here:

http://www.biomedcentral.com/1471-2350/13/12/prepub

## Supplementary Material

Additional file 1 **Real time reverse transcription assays for quantitative gene expression analysis**. Click here for file

Additional file 2 **Linkage disequilibrium around *BCL2L13, BID *and *MICAL3 *showing the eight transcript SNPs and the associated SNP rs2277831**. Click here for file

Additional file 3 **Primers and enzymes used for genotyping SNPs by RFLP analysis**. Click here for file

Additional file 4 **Sequenom assays for allelic expression imbalance analysis**. Click here for file

Additional file 5 **The Delta Ct values for the expression of *BCL2L13, MICAL3 *and *BID *in the cartilage of 32 OA patients**. Click here for file
