# Supplementary material for: Allelic expression analysis of the osteoarthritis susceptibility locus that maps to MICAL3
Source: BMC Med Genet. 2012 Mar 2;13:12. doi: 10.1186/1471-2350-13-12 (PMC3366887; doi:10.1186/1471-2350-13-12)
Supplement: Additional file 1 — Real time reverse transcription assays for quantitative gene expression analysis. [file 1471-2350-13-12-S1.PDF]

**Additional file 1 - Real time reverse transcription assays for quantitative gene expression analysis**

| Gene | Forward primer (5'-3') | Reverse primer (5'-3') | Probe (5'-3') |
|---|---|---|---|
| *18s* | CGAATGGCTCATTAAATCAGTTATGG | TATTAGCTCTAGAATTACCACAGTTATCC | TCCTTTGGTCGCTCGCTCCTCTCCC |
| *GAPDH* | ACATCGCTCAGACACCATG | TGTAGTTGAGGTCAATGAAGGG | AAGGTCGGAGTCAACGGATTTGGTC |
| *HPRT1* | TGCTGAGGATTTGGAAAGGG | ACAGAGGGCTACAATGTGATG | AGGACTGAACGTCTTGCTCGAGATG |
| *BCL2L13* | AGGTATGTCACGCCAAACTG | GGAATGTACACTGGAGACCAC | TCGTAGCAAAACCAGAGGCACCA |
| *BID* | ATTAACCAGAACCTACGCACC | TGACCACATCGAGCTTTAGC | CAGAAATGGGATGGACTGAACGGACA |
| *MICAL3* | GAACAAAGTGAAGTACATGGCG | TGGCAGAAGTAGCATGTGTC | ATCGGCATACGGAGACAGGGC |
