# Supplementary material for: Allelic expression analysis of the osteoarthritis susceptibility locus that maps to MICAL3
Source: BMC Med Genet. 2012 Mar 2;13:12. doi: 10.1186/1471-2350-13-12 (PMC3366887; doi:10.1186/1471-2350-13-12)

Additional file 2 - Linkage disequilibrium around *BCL2L13*, *BID* and *MICAL3* showing the eight transcript SNPs and the associated SNP rs2277831

The figures within boxes represent the r^2 value between SNPs.

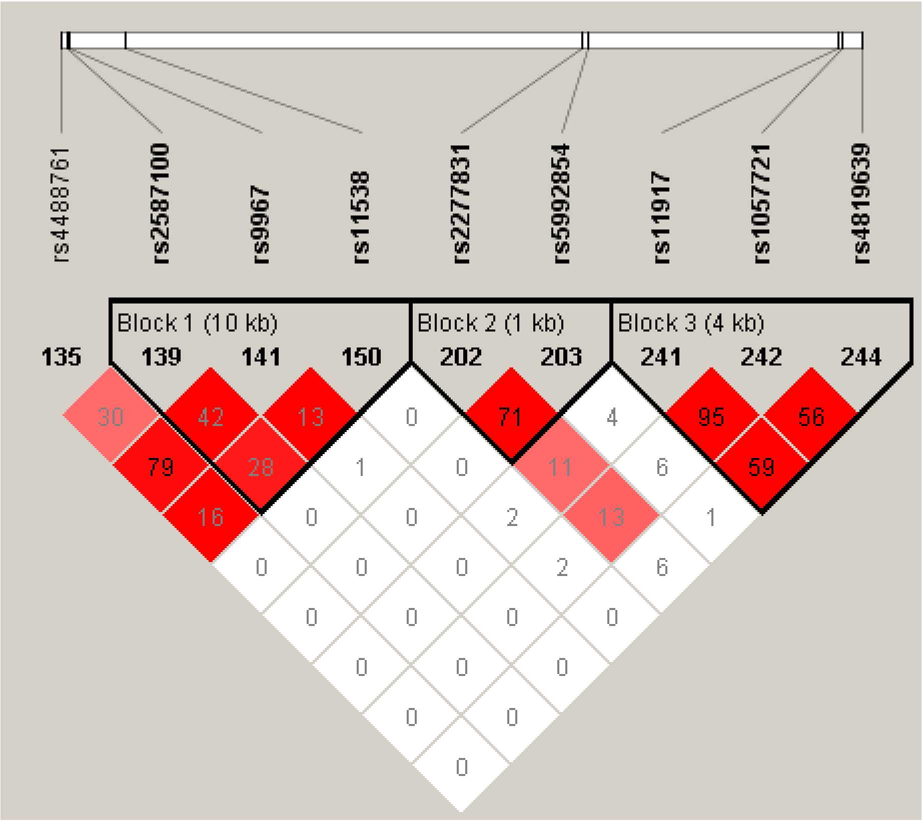

Supplement: Additional file 2 — Linkage disequilibrium around BCL2L13, BID and MICAL3 showing the eight transcript SNPs and the associated SNP rs2277831. [file 1471-2350-13-12-S2.PDF]
