# Supplementary material for: Allelic expression analysis of the osteoarthritis susceptibility locus that maps to MICAL3
Source: BMC Med Genet. 2012 Mar 2;13:12. doi: 10.1186/1471-2350-13-12 (PMC3366887; doi:10.1186/1471-2350-13-12)
Supplement: Additional file 3 — Primers and enzymes used for genotyping SNPs by RFLP analysis. [file 1471-2350-13-12-S3.PDF]

Additional file 3 - Primers and enzymes used for genotyping SNPs by RFLP analysis

SNP	Forward primer (5'-3')	Reverse primer (5'-3')	Enzyme
rs4488761	GCTTGTCCTTCGTTTCTAGG	CAGGATCCATTGCAATCTGC	AluI
rs2587100	GGTTTCTCAGCCTTGGCACTAGT	CTCTCGCTAAAGCAGTGGGTCTCAATAGGGGATGTTCACA	BstNI
rs9967	CCACTTGTGAGTGCAACTG	CCTAGCAAGAGACCCATAGAAC	BstNI
rs11538	CCTTGTGCTGGCATCAGAG	CCTACCCTAGAGACATGG	Tsp5091
rs2277831	GGATCACCTATGAAGAAAGAC	CAGGGATGGACAGCTAGTGG	MslI
rs5992854	GTCGGCCTTCTTCTTCTTGTC	GCTCTTTCACTTCATCCGAG	BstNI
rs11917	CCTCAAGCTCACTCCCAAGACCTG	CAGGAGAGGATTAGCTGTGC	HincII
rs1057721	TCAGCTATGGCTCTTGGGGTAAGGTGGGAGGTAAGCTGGATTG	CATAGTTCGGATCCTGAATGG	PstI
rs4819639	GGTCATGAAAGTGGGACAGGT	CCGTGGTCTTGTTTTGGGTTC	AluI
