# Supplementary material for: Allelic expression analysis of the osteoarthritis susceptibility locus that maps to MICAL3
Source: BMC Med Genet. 2012 Mar 2;13:12. doi: 10.1186/1471-2350-13-12 (PMC3366887; doi:10.1186/1471-2350-13-12)
Supplement: Additional file 4 — Sequenom assays for allelic expression imbalance analysis. [file 1471-2350-13-12-S4.PDF]

Additional file 4 - Sequenom assays for allelic expression imbalance analysis

Multi-plex	SNP	Forward primer (5'-3')	Reverse primer (5'-3')	Extension primer (5'-3')	Mass of extension primer (Da)
1	rs4488761	ACGTTGGATGTCTCCAACTGTGACCACTTC	ACGTTGGATGCTTTCTGTGTGCCAACTCTG	TTACCTGTGTCACTGTC	5127.3
1	rs2587100	ACGTTGGATGCCCAGAGATGACAAACGAAA	ACGTTGGATGAAGCAGTGGGTCTCAATAGG	GACATTGCCAAATGTCCC	5443.6
1	rs11917	ACGTTGGATGTGGTGTGTTTCTTTGTGAG	ACGTTGGATGAAGCACGACAACTTGGTTT	TGTGTAACTTTGTTTTTGCCGTT	7032.6
2	rs9967	ACGTTGGATGAACTCTGGCTAGAGAGACAC	ACGTTGGATGGGCTGAAGAGACCTCAGAAG	AAGGCAGGAGGATAACC	5277.5
2	rs1057721	ACGTTGGATGGCTTTTTAGTGGAGCAAGAG	ACGTTGGATGACACATCACCGTCAGCTATG	GGGAGGTAAGCTGGATTG	5659.7
2	rs4819639	ACGTTGGATGATGTCCCATCACGGTGCAG	ACGTTGGATGATTCTGCGTCCTGTTCCCTG	AGACAGGAGAGCAGCCACAG	6194.1
