# Supplementary material for: Allelic expression analysis of the osteoarthritis susceptibility locus that maps to MICAL3
Source: BMC Med Genet. 2012 Mar 2;13:12. doi: 10.1186/1471-2350-13-12 (PMC3366887; doi:10.1186/1471-2350-13-12)
Supplement: Additional file 5 — The Delta Ct values for the expression of BCL2L13, MICAL3 and BID in the cartilage of 32 OA patients. [file 1471-2350-13-12-S5.PDF]

Additional file 5 - The Delta Ct values for the expression of *BCL2L13*, *MICAL3* and *BID* in the cartilage of 32 OA patients

*Cart (H), hip cartilage; Cart (K), knee cartilage

Patient number	Sex	Age at surgery (years)	Tissue*	Genotype at rs2277831	Delta Ct		
					BCL2L13	*BID*	*MICAL3*
1	F	55	Cart (K)	GG	8.52	8.75	8.23
2	M	82	Cart (K)	GG	7.63	8.61	7.89
3	M	82	Cart (K)	GA	7.62	8.29	8.71
4	M	57	Cart (K)	GA	7.23	9.41	8.34
5	F	80	Cart (K)	GA	10.52	10.32	10.70
6	M	56	Cart (K)	GA	9.68	7.81	8.88
7	F	67	Cart (K)	GA	7.22	4.87	6.29
8	M	62	Cart (K)	GA	8.96	9.67	8.39
9	M	74	Cart (K)	GA	8.57	8.85	8.33
10	F	67	Cart (K)	GA	9.14	9.56	8.89
11	F	70	Cart (H)	GA	6.98	7.44	6.78
12	M	71	Cart (K)	AA	8.78	7.64	8.22
13	F	66	Cart (K)	AA	7.60	9.15	6.96
14	F	64	Cart (K)	AA	9.30	9.61	9.11
15	F	69	Cart (K)	AA	7.68	7.54	7.97
16	F	71	Cart (K)	AA	8.64	10.92	7.73
17	M	57	Cart (K)	AA	10.33	10.89	9.78
18	M	63	Cart (K)	AA	10.94	9.97	8.66
19	M	63	Cart (K)	AA	8.10	6.30	5.13
20	F	67	Cart (K)	AA	9.98	12.04	10.12
21	F	60	Cart (K)	AA	8.88	8.05	8.24
22	F	60	Cart (K)	AA	10.87	10.58	9.66
23	F	78	Cart (K)	AA	8.94	9.73	8.63
24	F	54	Cart (K)	AA	9.70	9.06	9.68
25	M	46	Cart (K)	AA	8.38	9.81	9.56
26	F	67	Cart (H)	AA	8.02	9.77	8.97
27	F	61	Cart (H)	AA	8.15	10.21	9.32
28	F	58	Cart (H)	AA	9.30	10.05	8.71
29	F	78	Cart (H)	AA	8.41	9.69	8.07
30	F	51	Cart (H)	AA	8.41	9.97	8.86
31	F	70	Cart (H)	AA	9.17	11.42	9.63
32	F	75	Cart (H)	AA	9.88	9.16	9.95
